# Lipidomics for Determining Giant Panda Responses in Serum and Feces Following Exposure to Different Amount of Bamboo Shoot Consumption: A First Step towards Lipidomic Atlas of Bamboo, Giant Panda Serum and Feces by Means of GC-MS and UHPLC-HRMS/MS

**DOI:** 10.3390/ijms231911544

**Published:** 2022-09-29

**Authors:** Chenglin Zhu, Xin Pan, Guo Li, Caiwu Li, Daifu Wu, Junni Tang, Yan Huang, Likou Zou, Luca Laghi

**Affiliations:** 1 College of Food Science and Technology, Southwest Minzu University, Chengdu 610041, China; 2 Research Center of Giant Panda National Park, Chengdu University of Technology, Chengdu 611059, China; 3 Key Laboratory of State Forestry and Grassland Administration on Conservation Biology of Rare Animals in the Giant Panda National Park, The China Conservation and Research Center for the Giant Panda, Dujiangyan 611800, China; 4 College of Resources, Sichuan Agricultural University, Chengdu 611130, China; 5 Department of Agricultural and Food Sciences, University of Bologna, 47521 Cesena, Italy

**Keywords:** bamboo, giant panda, lipidomics, GC-MS, UHPLC-HRMS/MS

## Abstract

Lipidic metabolites play essential roles in host physiological health and growth performance, serving as the major structural and signaling components of membranes, energy storage molecules, and steroid hormones. Bamboo, as wild giant pandas’ exclusive diet, is the main determinant of giant pandas’ lipidome, both as a direct source and through microbiota activity. Interestingly, the consumption of bamboo has attracted little attention from a lipidomic perspective. In the current study, we outline the lipidomic atlas of different parts of bamboo. By gas chromatography—mass spectrometry (GC-MS), we have been able to obtain the absolute quantification of 35 fatty acids pertaining to short chain fatty acids (8), medium chain fatty acids (6), long chain fatty acids (17), and very long chain fatty acids (4), while liquid chromatography coupled to high-resolution mass spectrometry (UHPLC-HRMS/MS) allowed us to obtain the relative quantification of another 1638 lipids. Among the fatty acids quantified in absolute terms, eight showed significantly distinct concentrations among different bamboo parts. Subsequently, we investigated how the giant panda’s serum and fecal lipidome adapt to the most important annual change in their diet, represented by the consumption of high amounts of bamboo shoots, typical of spring, the weight-gaining season. Five fatty acids were significantly altered in feces and two in serum, respectively, due to the different levels of bamboo shoot consumption. Furthermore, significant differences of the main bacteria strains were observed in feces between the two groups at the genus level, pertaining to *Streptococcus*, *Leuconostoc*, and *Vagococcus.* Correlations between giant panda fecal microbiome and lipidome were evaluated by Pearson correlation analysis. These findings suggest that a balanced diet, important for the overall lipidomic function and giant panda health, could be reached even in this remarkable case of a single food-based diet, by administering to the giant panda’s combinations of different parts of bamboo, with specific lipidome profiles.

## 1. Introduction

Improving the health of wild animals bred in zoos by adjusting their food profile is a far more challenging task than it is for farm animals. Farm animals are numerous and are routinely sacrificed for food, thus allowing invasive observations, and some of them have very short life cycles. Quoting Teare et al. [1], collecting information on how to optimize diets in zoos is like trying to imagine the forest from cross-sections of individual trees. Therefore, together with the transformation of zoos from animal expositions to catalysts for the preservation of endangered species, great efforts have been devoted to creating and populating databases such as MedARKS (the Medical Animal Record Keeping System), with reference ranges for physiological values in captive wildlife [2] or information about optimal nutrition.

The difficulty of diet optimization through the collection of physiological data reaches its acme when dealing with giant pandas (*Ailuropoda melanoleuca*). In fact, their diet is constituted exclusively by bamboo, but their short digestive tract and enzymatic profile, typical of carnivores, is inadequate for breaking down bamboo stalks efficiently.

Very interestingly, some sort of diet variability is offered by the various parts of the bamboo, which have a markedly different nutritional profile. In particular, shoots are far richer in carbohydrates and proteins than stems and leaves, so much that they are the sole bamboo parts that can be employed to wean juvenile giant pandas for their introduction into their natural habitat [3]. In addition, giant pandas are known to primarily consume shoots in spring, leaves in summer and fall, and stems in winter and early spring, before new shoots are available again [4]. This is well documented in the wild, where giant pandas cyclically move back and forth between portions of their habitat with higher and lower elevations in search of specific parts of the bamboo [5]. Interestingly, the parts of the bamboo preferred by captive giant pandas mimic this trend throughout the year [3].

Until now, there is no doubt that most of the attention has been devoted to gut microbiota in connection to the seasonal shifts in the consumption of different bamboo parts. For instance, Williams et al. found that leaf preference throughout the year corresponded to significant shifts in the densities of total aerobes, *streptococci*, *lactobacilli* and *bacteroides* spp. [6]. Wu et al. and Wang et al. found a more diverse gut microbiome during the bamboo shoot stage in comparison to the leaf stage [7,8]. In addition, the latter authors noticed that the higher flavonoid content in the leaves led to a lower virulence factor and to a higher presence of cellulose-degrading species [8].

Recent investigations from some of the authors of the present work demonstrated that a wealth of additional information can be obtained on giant pandas’ health status through metabolomic investigations. Unfortunately, those studies have been limited to water soluble molecules, thus neglecting a key portion of the metabolome, from a health perspective, represented by the lipidome. Lipidomic features are essential for physiological health and growth performance, serving as the major structural and signaling regulatory components of membranes, energy storage molecules, and steroid hormones [9]. This is why the dysregulation of lipid metabolism is considered a driver of metabolic disease [10]. When dealing with the giant panda’s lipidome, a complete metabolomic investigation cannot neglect the composition of the various bamboo parts themselves. In fact, even though fatty acids, especially short-chain fatty acids (SCFAs), are mainly produced by gut microbiota, it is worth noticing that the lipid fraction of bamboo is transferred to the giant panda gut directly.

In order to fill the mentioned gaps, in the present study, for the first time, we tried to set up a lipidomic atlas of different bamboo parts by means of gas chromatography coupled with mass spectrometry (GC-MS) and liquid chromatography coupled to high-resolution mass spectrometry (UHPLC-HRMS/MS), which are well-established techniques for identifying and quantifying fatty acids in samples [11,12]. In addition, quantitative information on giant panda feces and serum lipid fractions were obtained by comparing their metabolome after administering two levels of bamboo shoots, one typical of the diet normally followed throughout the year and one much higher, typical of the spring—the weight-gaining season.

## 2. Results

### 2.1. A Lipidomic Atlas of Bamboo

We had the opportunity to obtain the absolute quantification of 8 short chain fatty acids (SCFAs), 6 medium chain fatty acids (MCFAs), 13 long chain fatty acids (LCFAs), and 4 very long chain fatty acids (VLCFAs) by means of targeted GC-MS approach. In addition, to obtain the richest possible information on the lipidome of bamboo, we performed an untargeted lipidomic investigation by means of UHPLC-HRMS/MS. This allowed us to acquire the relative concentrations of further 1638 lipids (862 in negative mode and 776 in positive mode) in the bamboo samples observed. The entire concentration values are reported in the supporting material. Among the fatty acids quantified in absolute terms, eight showed statistically significant differences among the three bamboo parts investigated, as shown in Table 1.

**Figure 1:**
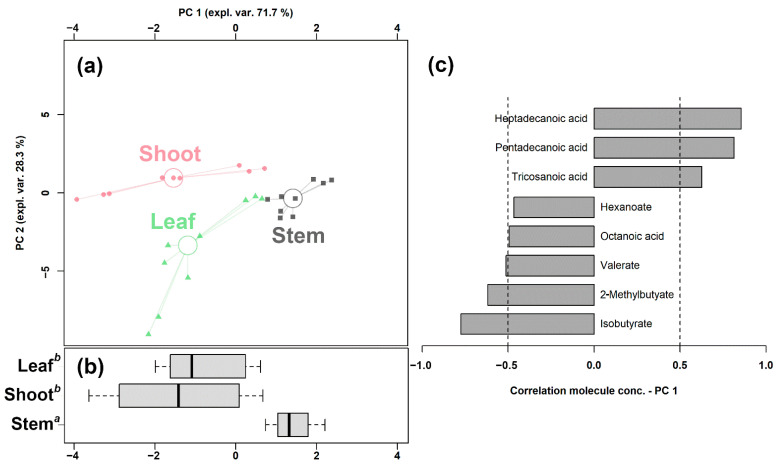
rPCA model built on the concentration of the fatty acids that showed a statistically significant difference among shoots, leaves, and stems. In the scoreplot (**a**), samples from the three groups are represented with circles, triangles, and squares, respectively. The wide, empty circles represent the median of the samples of various samples. The position of the samples along PC 1 is summarized in boxplot (**b**). The loading plot (**c**) reports the correlation between the concentration of each substance and its importance over PC 1.

In order to underline the overall underlying trends among the bamboo parts, we set up an rPCA model based on the significantly changed fatty acids (Figure 1).

Its first component, representing 71.7% of the variance thus expressed, indeed significantly summarized the peculiarities of the different bamboo parts (*p* < 0.05), with shoots and stem samples appearing at low and high PC scores, respectively, and leaf samples at intermediate values, closer to the shoots. Furthermore, a PLS-DA model and a heatmap were set up to summarize the differences among the three groups in the untargeted data obtained through UHPLC-HRMS/MS. As shown in Figure S4, the PLS-DA model exhibits a good ability to discriminate the groups from each other (R^2^Y > 0.5 and Q^2^ > 0.5). The heatmap of Figure S5 allows us to visually appreciate that bamboo shoots were characterized by higher levels of SCFAs and MCFAs, while LCFAs and VLCFAs were more abundant in leaves and stems.

### 2.2. Normal and High Bamboo Shoot Consumption Affect the Lipidomic Features of Giant Pandas

In giant panda feces, we were able to absolutely quantify 7 SCFAs, 5 MCFAs, 15 LCFAs, and 4 VLCFAs by means of GC-MS. Among these fatty acids, the concentrations of 2 SCFAs (propanoate and formate), 1 MCFAs (decanoic acid), 1 LCFA (palmitoleic acid), and 1 VLCFAs (tricosanoic acid) showed significant differences between Normal and High shoots intake Groups, as shown in Table 2. Moreover, four LCFAs and one VLCFA, namely arachidic acid, *cis*-11-eicosenoic acid, *cis*-11,14-eicosadienoic acid, *cis*-5,8,11,14-eicosatetraenoic acid, and lignoceric acid, showed a trend toward significance (0.05 < *p* < 0.1).

**Figure 2:**
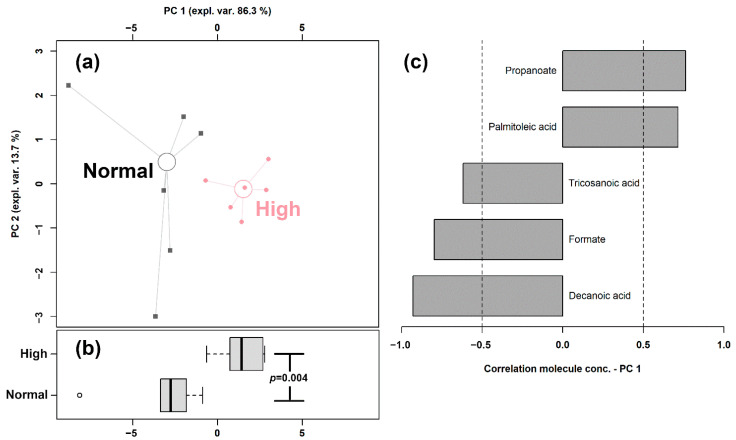
rPCA model built on the concentration of the fatty acids quantified in giant panda feces that showed a statistically significant difference between Normal and High shoots intake groups. In the scoreplot (**a**), samples from the two groups are represented with circles and squares, respectively. The wide, empty circles represent the median of the samples of various subjects. The position of the subjects along PC1 is summarized in boxplot (**b**). The loading plot (**c**) reports the correlation between the concentration of each substance and its importance over PC 1.

In order to highlight the overall trend of the data, we set up an rPCA model, shown in Figure 2, based on the concentration of these fatty acids.

The first principal component of the two needed to robustly describe the samples and significantly summarize the peculiarities connected to the amount of bamboo shoots consumed (*p* < 0.05), with samples from the Normal and High intake groups appearing as low and high scores, respectively. The high concentrations of propanoate and palmitoleic acid represented the main features of the feces of giant pandas consuming high amounts of shoots, while higher concentrations of tricosanoic acid, formate, and decanoic acid characterized the Normal shoots intake group.

**Figure 3:**
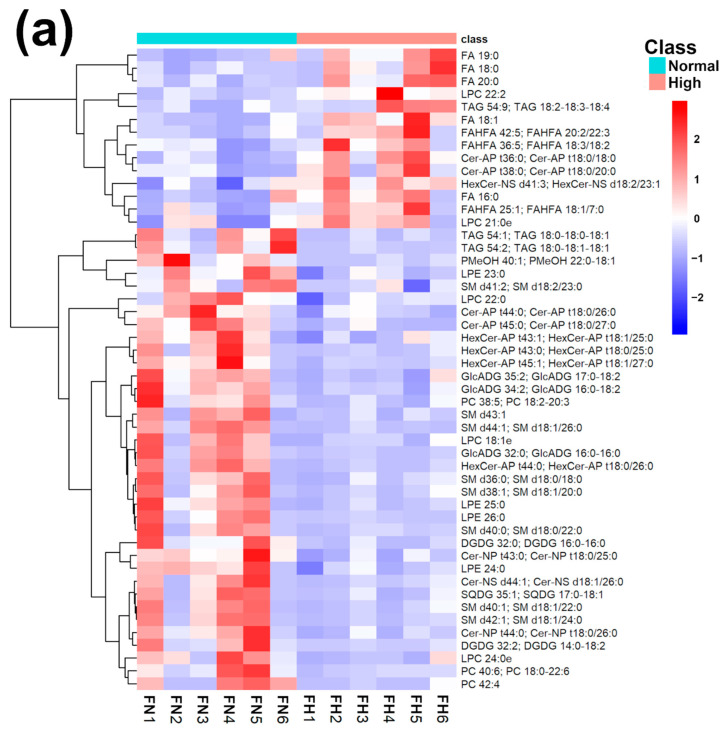

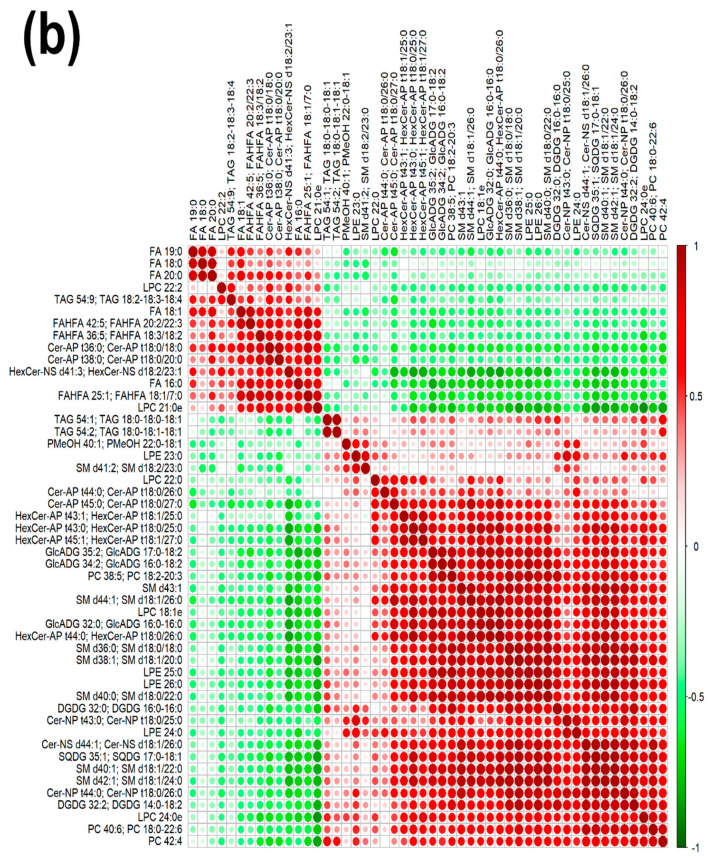

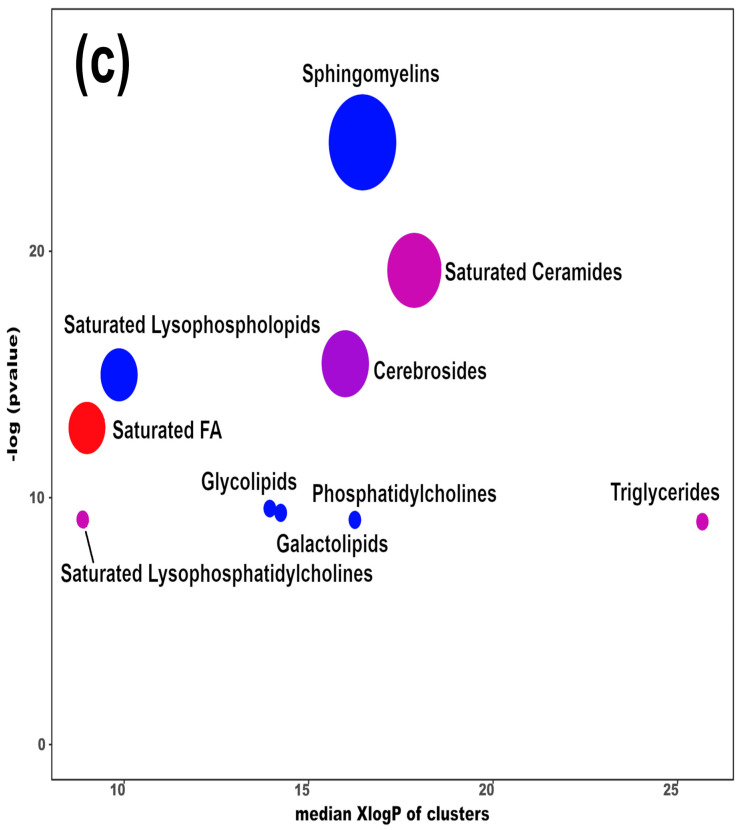
Heatmap (**a**), correlation plot (**b**), and ChemRICH set enrichment statistics plot (**c**) of the concentration of the fatty acids quantified by UHPLC-HRMS/MS in giant panda feces, which showed a statistically significant difference between groups. In the ChemRICH set enrichment statistics plot, each node reflects a significantly altered cluster of molecules. Enrichment *p*-values are calculated through the Kolmogorov–Smirnov test. Node sizes represent the total number of metabolites in each cluster set. The node color scale shows the proportion of increased (red) or decreased (blue) compounds in the High bamboo shoot consumption group compared to the Normal group. Purple-color nodes have both increased and decreased molecules.

In order to underline the entire lipidome’s features of giant panda feces affected by different intakes of bamboo shoot, PLS-DA, heatmap, correlation plot, and ChemRICH set enrichment statistics plot were applied to the relative, untargeted quantification of lipids obtained through UHPLC-HRMS/MS, as shown in Figure 3 and Figure S6.

The established PLS-DA model exhibited a good ability to discriminate groups from each other, as shown in Figure S6, which is suitable for explaining the differences among groups (R^2^Y > 0.5) and prediction (Q^2^ > 0.5). Furthermore, as shown in Figure 3a,b, the concentrations of saturated fatty acids were more abundant when giant panda’s consumed a higher level of bamboo parts, and such fatty acids showed positive correlations. Moreover, the result of the ChemRICH set enrichment statistics plot confirmed the above trends (Figure 3c). In detail, fecal fatty acid clusters increased in the high bamboo shoot consumption group was saturated fatty acids, while the following were decreased in the normal bamboo shoot consumption group, namely sphingomyelins, saturated lysophospholipids, glycolipids, galactolipids, and phosphatidylcholines.

In terms of serum samples, a lower number of fatty acids were quantified, compared to fecal samples, pertaining to 5 SCFAs, 4 MCFAs, 13 LCFAs, and 1 VLCFAs. Among the quantified molecules, only two showed a significant difference between the two groups, namely undecanoic acid (Normal Group: 0.75 ± 0.42 mmol/L; High Group: 0.26 ± 0.16 mmol/L; *p*-value = 0.043) and heptadecanoic acid (Normal Group: 7.90 ± 1.06 mmol/L; High Group: 5.81 ± 0.61 mmol/L; *p*-value = 0.015). Meanwhile, lauric acid, pentadecanoic acid, and *cis*-13-docosenoic acid showed a trend toward significance (0.05 < *p* < 0.1).

**Figure 4:**
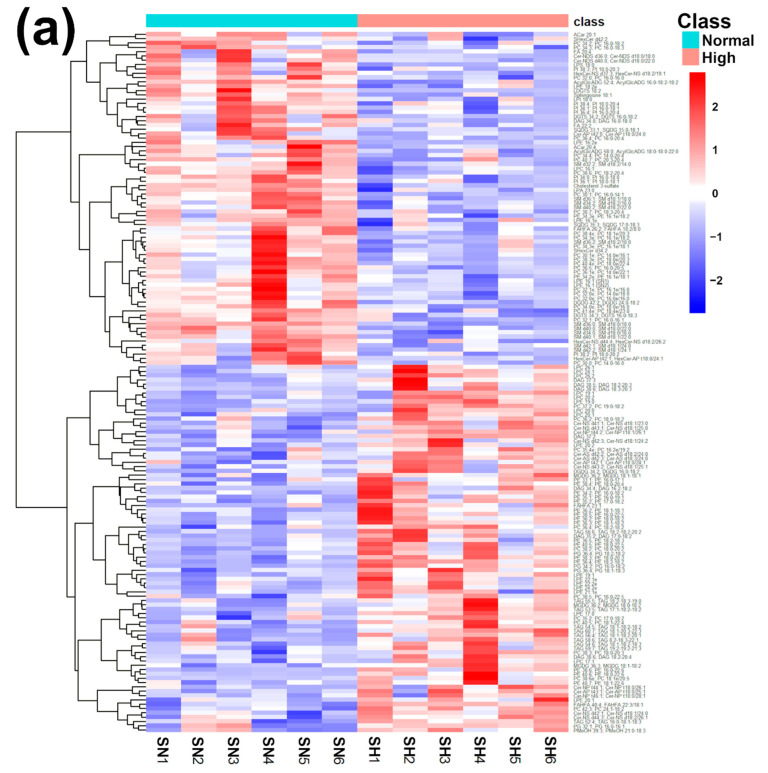

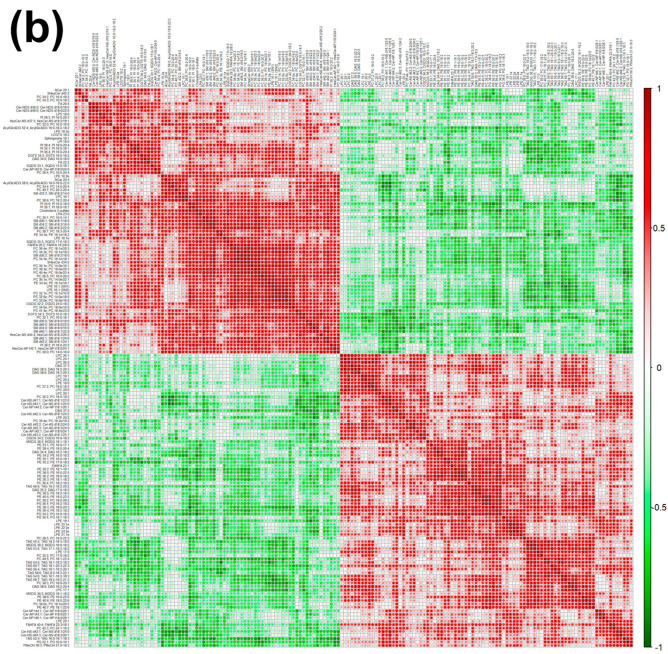

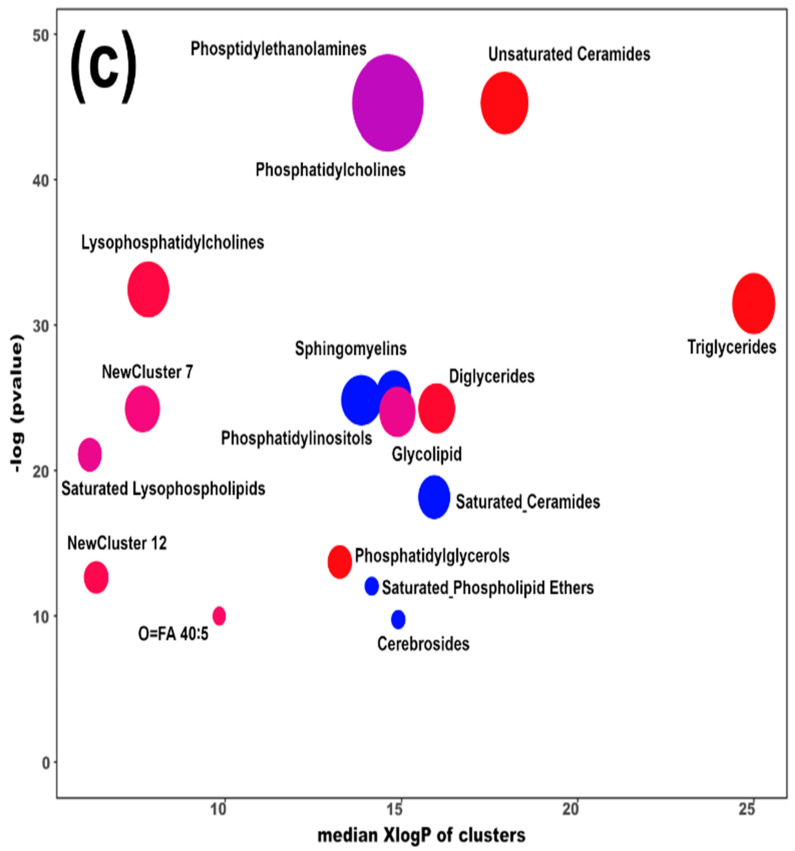
Heatmap (**a**), correlation plot (**b**), and ChemRICH set enrichment statistics plot (**c**) of the concentration of the fatty acids quantified by UHPLC-HRMS/MS in giant panda serum samples, which show a statistically significant difference between groups. In the ChemRICH set enrichment statistics plot, each node reflects a significantly altered cluster of molecules. Enrichment *p*-values are calculated through the Kolmogorov–Smirnov test. Node sizes represent the total number of metabolites in each cluster set. The node color scale shows the proportion of increased (red) or decreased (blue) compounds in the High bamboo shoot consumption group compared to the Normal group. Purple-color nodes have both increased and decreased molecules.

Similar to fecal samples, a set of multivariate investigations made by PLS-DA, heatmap, correlation plot, and ChemRICH set enrichment statistics plot was applied to the relative quantities of lipids obtained through untargeted UHPLC-HRMS/MS, aiming to highlight the key lipid pathways triggered by a high intake of bamboo shoots, as shown in Figure 4 and Figure S7.

The established PLS-DA model exhibited a good ability to discriminate the groups from each other (R^2^Y > 0.5 and Q^2^ > 0.5), as shown in Figure S7. Furthermore, as shown in Figure 4a,b, the concentrations of unsaturated ceramides were more abundant when giant pandas consumed a high level of bamboo shoots. The result of the ChemRICH set enrichment statistics plot was coherent with these trends, as shown in Figure 4c. In detail, fatty acids clusters increased in the High bamboo shoot consumption group were triglycerides, unsaturated ceramides, diglycerides, lysophosphatidylcholines, and phosphatidylglycerols, while the following were decreased in the Normal group, namely sphingomyelins, saturated ceramides, saturated phospholipid ethers, and cerebrosides.

### 2.3. Microbial Community and Their Association with Lipidomic Profiles

For 16S rRNA gene sequencing, *Firmicutes* (Normal Group: 74.4%, High Group: 66.5%), *Proteobacteria* (Normal Group: 24.5%, High Group: 31.9%), and *Bacteroidota* (Normal Group: 0.6%, High Group: 0.7%) were the major phyla in Giant panda feces. None of the phyla showed significant differences between the two groups. At the genus level, *Streptococcus* (Normal Group: 58.9%, High Group: 37.7%), *Escherichia* (Normal Group: 21.5%, High Group: 24.6%), and *Clostridium* (Normal Group: 6.1%, High Group: 10.0%) were the three most represented genera in Giant panda fecal samples. *Streptococcus* was significantly higher in the Normal group. On the contrary, *Leuconostoc* and *Vagococcus* were significantly more abundant in the High bamboo shoot consumption group. Pearson correlation analysis showed that the amount of *Streptococcus* was significantly positively correlated with concentration of formate (*p* = 0.03, *r* = 0.65), while *Vagococcus* exhibited negative correlation with concentration of formate (*p* = 0.04, *r* = −0.63). Moreover, the level of propanoate was significantly positively correlated with *Vagococcus* (*p* = 0.002, *r* = 0.83).

## 3. Discussion

In the present study, we desired to outline through a lipidomic investigation, key features of bamboo parts that could be employed to improve the health of giant pandas through diet, even if based on a single food. On one side, we sought to obtain the widest possible atlas of molecules constituting the lipidome of bamboo’s leaves, stems, and shoots. On the other side, we focused on the richest nutritional source, the shoots, with the aim of highlighting how giant pandas’ lipidome was affected by normal and high shoots intake, typical of the mating season.

In giant panda feces, we found that the concentrations of two SCFAs (propanoate and formate), one MCFAs (decanoic acid), one LCFAs (palmitoleic acid), and one VLCFAs (tricosanoic acid) were significantly affected by the intake of bamboo’s shoots. Interestingly, the variations of the two SCFAs showed an opposite trend, with propionate higher and formate lower in the animals consuming high amounts of bamboo shoots. SCFAs are considered as the end-product of the microbial digestion of carbohydrates. The metabolism of propionic acid begins with its conversion to propionyl coenzyme A. Since propionic acid has three carbons, propionyl-CoA cannot directly enter either beta-oxidation or the citric acid cycles. Propionate’s concentration is higher in the feces of giant pandas consuming high amounts of bamboo shoots could, therefore, be coherent with the higher intake of carbohydrates, and in turn, energy granted by the shoots. Formate could be involved too in the energy metabolism through fermentation of carbohydrates and degradation of amino acids [13]. Anyway, its lower concentration in the feces of giant pandas consuming higher amounts of shoots could reflect its low concentration in the shoots themselves, as highlighted by our metabolomic investigation of bamboo’s parts. Moreover, 16S rRNA analysis showed that the content of formate in feces was positively associated with the relative abundance of *Streptococcus* and negatively associated with *Vagococcus*. Propanoate concentration was positively correlated with the relative abundance of *Vagococcus*. There is no doubt about the interpretation of the positive correlation between formate and *Streptococcus*, due to its formate production ability [14]. Focusing on *Vagococcus*, it was found in chicken’s feces and fermented milk, while its biological functions still need to be further investigated [15]. Integrated with fecal microbiota profile, we could speculate that higher bamboo shoot consumption could affect giant panda gut microbiota, and, in turn, could contribute to the variations of SCFAs.

Decanoic acid, as one of MCFAs with ten carbons, was successfully quantified in the feces of several mammals, including humans and pigs, where its antimicrobial properties against *Escherichia coli* was highlighted [16,17]. Interestingly, Wilson et al. found that the level of decanoic acid was elevated in female giant pandas’ urine during the breeding season, triggering the sexual responses in males [18]. This piece of information suggests that the lower amount of decanoic acid in giant pandas fed with high amounts of shoots could be related to hormonal perturbations, possibly gut microbiota related.

Palmitoleic acid, as one of the LCFAs, has been found in humans to play a key role in case of energy metabolism imbalances [19]. Specifically, Schwiertz et al. reported that palmitoleic acid increased after a high-fat, low-carbohydrate diet, associated with negative effects on gut microbiota and microbial metabolites [20]. This suggests that the higher level of palmitoleic acid evidenced by the present study in connection with a high intake of bamboo shoots could be linked to the perturbations of gut microbiota. In this respect, a contribution may come from the palmitoleic acid present in the shoots themselves, more concentrated than in stem and leaf, even if not significantly. A confirmation that the composition of the food may reflect directly in the fecal lipidome may come from tricosanoic acid, less concentrated in shoots than in leaves and stems and indeed less concentrated in the feces of animals fed with high amounts of bamboo shoots.

In the serum samples, two fatty acids showed significant differences in connection with high amounts of shoots consumed, namely undecanoic and heptadecanoic acids. Both were more concentrated in the group fed with normal levels of shoots. Undecanoic acid was quantified in bamboo too, with higher levels in leaves and stem. Therefore, its trend may be a further confirmation that the lipidome profile of food may translate directly into that of the giant pandas.

Combined with giant panda serum and bamboo lipidomic data, it is worth noticing the odd chain saturated fatty acids (OCSFAs) quantified in both samples, namely pentadecanoic acid and heptadecanoic acid (FA: C15:0 and C17:0). In the current study, we found that giant panda consumed a large amount of bamboo shoots could decrease the level of heptadecanoic acid in serum. Furthermore, the concentrations of the two OCSFAs showed statistically significant differences among different bamboo parts. In detail, bamboo shoots contained lower levels of heptadecanoic acid than leaves and stems. Odd chain saturated fatty acids are produced by α-oxidation in peroxisomes, de novo lipogenesis, from the diet and by gut microbiota. Although present at low concentrations, epidemiological studies show that higher circulating levels of odd chain saturated fatty acids (FA: C15:0 and C17:0) are associated with lower risks of metabolic diseases [21].

## 4. Materials and Methods

### 4.1. Bamboo Samples

A total of 27 fresh bamboo samples were collected, equally representing leaves, shoots, and stems, from a mix of the three main bamboo species (*Chimonobambusa neopurpurea, Pleioblastus amarus*, and *Bashania fargesii*) growing in the China Conservation and Research Center for the Giant Panda.

### 4.2. Giant Pandas and Their Diets

A total of 12 healthy captive giant pandas from the China Conservation and Research Center for the Giant Panda were the subject of this study. For ten days, six of them were fed with 7.5 Kg/d of bamboo shoots, so to mimic a normal consumption (Normal Group) maintained throughout the year. The other six were fed with 15 Kg/d of bamboo shoots, so to mimic a high consumption typical of the spring (High Group). The remaining diet was identical between the two groups, constituted by bamboo stems and leaves, added with fruits, vegetables, and corn/wheat concentrates.

### 4.3. Feces and Serum Sample Collection

All sample collection protocols in this study were approved by the China Conservation and Research Center for the Giant Panda. The experimental procedures were in full compliance with the current Chinese laws on animal welfare and research.

A total of twenty-two samples of feces and serum were collected after the diets with normal and high amounts of shoots. Ten samples (five feces and five serum samples) were obtained from the Normal Group, and twelve samples (six feces and six serum samples) from the High Group. Fecal samples were collected immediately after defecation in sterilized plastic bags. Blood samples were collected from giant pandas during routine physical examinations by experienced staff veterinarians at the China Conservation and Research Center for the Giant Panda. All the above-collected samples were kept cold with dry ice during transport to the laboratory and stored at −80 °C until analysis.

### 4.4. GC-MS Analysis

In terms of SCFAs, an amount of 100 mg bamboo sample, 100 mg feces sample, and 100 μL serum sample was taken for GC-MS analysis, respectively. The workflow of sample preparation was reported in Figure S1. The detailed sample preparation procedures and instrumental analysis parameters were reported in the supporting materials.

As for MCFAs, LCFAs, and VLCFAs quantifications, we took 50 mg of feces and bamboo powder samples and 20 μL thawed serum sample, respectively, for GC-MS analysis. The workflow of sample preparation was reported in Figure S2. The detailed sample preparation procedures and instrumental analysis parameters were reported in the supporting materials.

### 4.5. UHPLC-HRMS/MS Analysis

In order to obtain an overall view of lipidomic profiles, 40 mg bamboo, 100 mg feces, and 50 μL serum samples were collected respectively for untargeted lipidomic investigations by means of UHPLC-HRMS/MS. The workflow of sample preparation was reported in Figure S3. The detailed sample preparation procedures and instrumental analysis parameters were reported in the supporting materials.

### 4.6. Molecules Identification and Quantification

**Data of GC-MS:** The GC-MS analysis software MSD Chem Station of Agilent (version E.02.02.1431, Agilent Technologies, Inc., Shanghai, China) was used to view the sample spectrum, and the default parameters were used to integrate the 35 fatty acids in the sample and the standard curve, and the standard curve was generated and analyzed, as shown in supporting materials. Quantitative analysis, colleague-assisted manual inspection to ensure the qualitative and quantitative accuracy of each compound.

**Data of UHPLC-HRMS/MS:** The raw data of UHPLC-HRMS/MS were firstly transformed to mzXML format by ProteoWizard and then processed by XCMS and CAMERA packages in the R software platform. In XCMS package, the peak picking (method = centWave, ppm = 5, peakwidth = c (5,20), snthresh = 10), alignment (bw = 6 and 3 for the first and second grouping, respectively), and retention time correction (method = obiwarp) were conducted. In the CAMERA package, the annotations of isotope peak, adducts, and fragments were performed with default parameters. The accurate m/z of precursors and product ions were matched against the LipidBlast database. The threshold of matching similarity is larger than 80%. A manual curation strategy was performed to improve the quality of lipid identification.

### 4.7. DNA Extraction and Amplicon Sequencing

Following the method of Jin et al. [22], 50 g of each fresh feces were prepared for DNA extraction and amplicon sequencing. In brief, the total microbial genomic DNA was extracted by means of the MoBio Power Fecal DNA Isolation Kit (MoBio Laboratories, Inc., Carlsbad, CA, USA) according to the manufacturer’s instructions. Successful DNA isolation was confirmed by agar gel electrophoresis. Bacterial amplicon libraries were prepared by amplifying the V4 region of 16S rRNA. Sequencing was performed using a 250-bp paired-end sequencing protocol on the Illumina MiSeq 2500 platform (Illumina, San Diego, CA, USA). The extraction kits and reagents in this study were used as negative (blank) controls, and no contaminant sequences were detected.

### 4.8. Statistical Analysis

**Data of GC-MS:** The statistical analysis was limited only to the molecules quantified in each group of samples. Variations in fatty acids whose concentration varied between the Normal and High shoots consumption groups were investigated by means of the Student’s *t*-test. Differences in the composition of bamboo parts were investigated by the ANOVA test followed by the Tukey HSD test. In detail, the aov function of the R package “stats” [23] was applied to the parameters expressed as ranks [24]. Prior to analysis, not-normally distributed data were brought to normality, following Box and Cox [25]. For each analysis, a significance limit *p* < 0.05 was accepted. To highlight the overall trends characterizing the samples, we built robust principal component analysis (rPCA) models [26] on the molecules accepted by the univariate analyses described above. For each model, we calculated the scoreplot, the projection of the samples in the PC space, tailored to highlight the underlying structure of the data. Besides, we calculated the correlation plot, according to Pearson, relating the concentration of each variable to their importance over each component of the model.

**Data of UHPLC-HRMS/MS:** The statistical analysis was limited only to the molecules quantified in each group of samples. The data were normalized against total peak areas before performing univariate and multivariate statistics. For univariate statistical analysis, the normalized data were calculated by a Student’s *t*-test. For multivariate statistical analysis, the normalized data were imported to SIMCA software (version 14.1, AB Umetrics, Umeå, Sweden), where the data were preprocessed by uv scaling and mean centering before performing PLS-DA. To avoid model over-fitting, a default 7-round cross-validation was performed, to determine the optimal number of principal components. ChemRICH analysis was performed based on the online ChemRICH software (http://chemrich.fiehnlab.ucdavis.edu, accessed on 25 June 2022).

**Data on fecal microbiota:** Paired-end reads were merged using FLASH (V1.2.7), and quality filtering of reads were performed via QIIME (V1.9.1). Chimera sequences were removed using the UCHIME algorithm. Tags were assigned to each sample with unique barcodes. Operational taxonomic units (OTUs) were clustered using UPARSE (V7.0.1001) using a 97% similarity threshold. Sequences for each OTU were referenced against the SILVA and UNITE databases to assign taxonomic classification. Kruskal–Wallis sum-rank test was utilized to analyze the differences in the microbiome profile between groups. The Pearson correlation analysis was used to assess the correlation between giant panda fecal microbiome and lipidome.

## 5. Conclusions

To the best of our knowledge, this is the first attempt to systematically characterize the lipidomic profiles of different bamboo parts, giant panda feces and serum, by means of GC-MS and UHPLC-HRMS/MS. In the current study, the widest possible atlas of molecules constituting the lipidome of bamboo’s leaves, stems, and shoots was obtained. Furthermore, distinct lipidomic profiles among bamboo parts were investigated, namely higher levels of SCFAs and MCFAs in bamboo shoots, while LCFAs and VLCFAs were more abundant in leaves and stems. Subsequently, we illustrated that giant panda serum and fecal lipidome could be altered so as to adapt to the most important annual change in their diet, represented by the consumption of high amounts of bamboo shoots, typical of spring, the weight-gaining season. Such variations could attribute to distinct lipidomic profiles of different bamboo shoot consumption, as well as the perturbations of gut microbiota. These findings suggest that a balanced diet, important for the overall lipidomic function and giant panda health, could be reached even in this remarkable case of a single food-based diet, by administering to the giant panda’s combinations of different parts of bamboo, with specific lipidome profiles.

## Figures and Tables

**Table 1:** Concentrations (mean ± SD, mmol/g) of the fatty acids quantified in absolute terms, which showed significant differences among the bamboo parts observed.

	Stem	Shoot	Leaf
Isobutyrate	1.17 ± 0.39 ^b,^*	1.71 ± 0.63 *^a,b^*	2.06 ± 1.03 *^a^*
2-Methylbutyate	1.12 ± 0.40 *^b^*	2.54 ± 0.48 *^a^*	1.42 ± 0.67 *^b^*
Valerate	0.39 ± 0.11 *^b^*	0.35 ± 0.20 *^b^*	0.88 ± 0.31 *^a^*
Hexanoate	3.16 ± 1.83 *^b^*	3.95 ± 2.46 *^b^*	24.30 ± 15.90 *^a^*
Octanoic acid	0.55 ± 0.07 *^b^*	0.97 ± 0.33 *^a^*	0.43 ± 0.18 *^b^*
Pentadecanoic acid	4.12 ± 0.77 *^a^*	1.83 ± 0.63 *^b^*	1.77 ± 1.09 *^b^*
Heptadecanoic acid	5.18 ± 1.36 *^a^*	3.36 ± 1.18 *^b^*	3.08 ± 1.31 *^b^*
Tricosanoic acid	2.88 ± 0.62 *^a^*	2.19 ± 0.52 *^a,b^*	1.97 ± 0.43 *^b^*

* For each molecule, the comparisons among the groups are represented by a compact letter display, where mean values followed by a common superscript identify no significant differences.

**Table 2:** Fecal fatty acids whose concentrations (mean ± SD, mmol/g) were significantly altered in connection with normal and high intake of bamboo shoots.

	Normal Group	High Group	*p*-Value	Trend
Formate	102.00 ± 103.00	23.30 ± 15.20	0.035	↓
Propanoate	2.45 ± 1.38	10.60 ± 5.20	0.005	↑
Decanoic acid	1.08 ± 0.52	0.48 ± 0.10	0.013	↓
Palmitoleic acid	2.52 ± 2.08	6.24 ± 2.36	0.015	↑
Tricosanoic acid	1.66 ± 0.11	1.29 ± 0.35	0.047	↓

## Data Availability

Not applicable.

## Supplementary Materials

The following supporting information can be downloaded at: https://www.mdpi.com/article/10.3390/ijms231911544/s1.
