# Supplementary material for: Lipidomics for Determining Giant Panda Responses in Serum and Feces Following Exposure to Different Amount of Bamboo Shoot Consumption: A First Step towards Lipidomic Atlas of Bamboo, Giant Panda Serum and Feces by Means of GC-MS and UHPLC-HRMS/MS
Source: Int J Mol Sci. 2022 Sep 29;23(19):11544. doi: 10.3390/ijms231911544 (PMC9569542; doi:10.3390/ijms231911544)
Supplement: Supplementary file 1 [file ijms-23-11544-s001.zip › ijms-1949955-supplementary.pdf]

Supplementary Materials

Full title: Lipidomics for Determining Giant Panda Responses in Serum and Feces Following Exposure to Different Amount of Bamboo Shoot Consumption: A first step towards lipidomic atlas of bamboo, giant panda serum and feces by means of GC-MS and UHPLC-HRMS/MS

Chenglin Zhu[1, #], Xin Pan[2, #], Guo Li[3], Caiwu Li[3], Daifu Wu[3], Junni Tang[1], Yan Huang[3], Likou Zou[4, *], Luca Laghi[5]

[1]College of Food Science and Technology, Southwest Minzu University, Chengdu, Sichuan 610041, China

[2]Research Center of Giant Panda National Park, Chengdu University of Technology, Chengdu, Sichuan 611059, China

[3]Key Laboratory of State Forestry and Grassland Administration on Conservation Biology of Rare Animals in the Giant Panda National Park, the China Conservation and Research Center for the Giant Panda, Dujiangyan, Sichuan, 611800, China.

[4]College of Resources, Sichuan Agricultural University, Chengdu, Sichuan 611130, China.

[5]Department of Agricultural and Food Science, University of Bologna, 47521 Cesena, Italy.

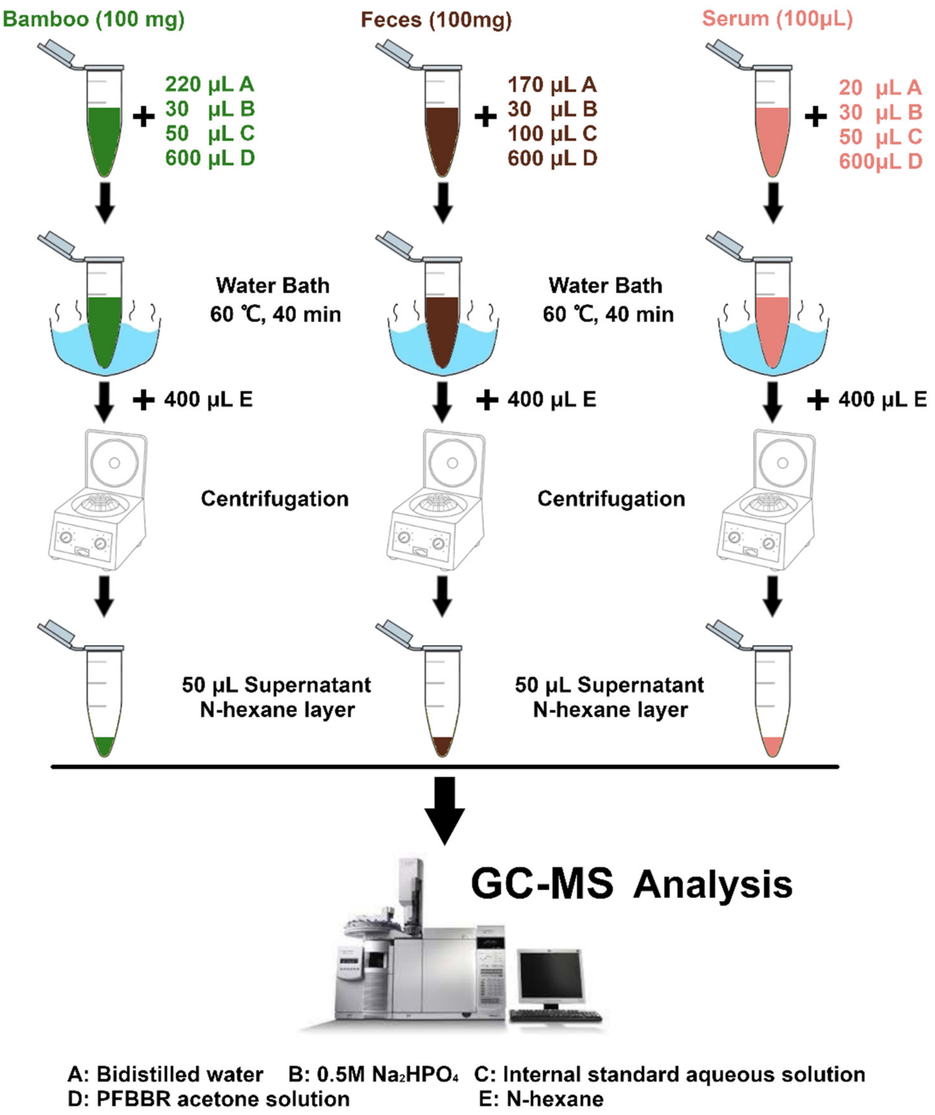

Figure S1. The workflow of sample preparation of bamboo, giant panda feces and serum using for SCFAs quantifications by means of GC-MS.

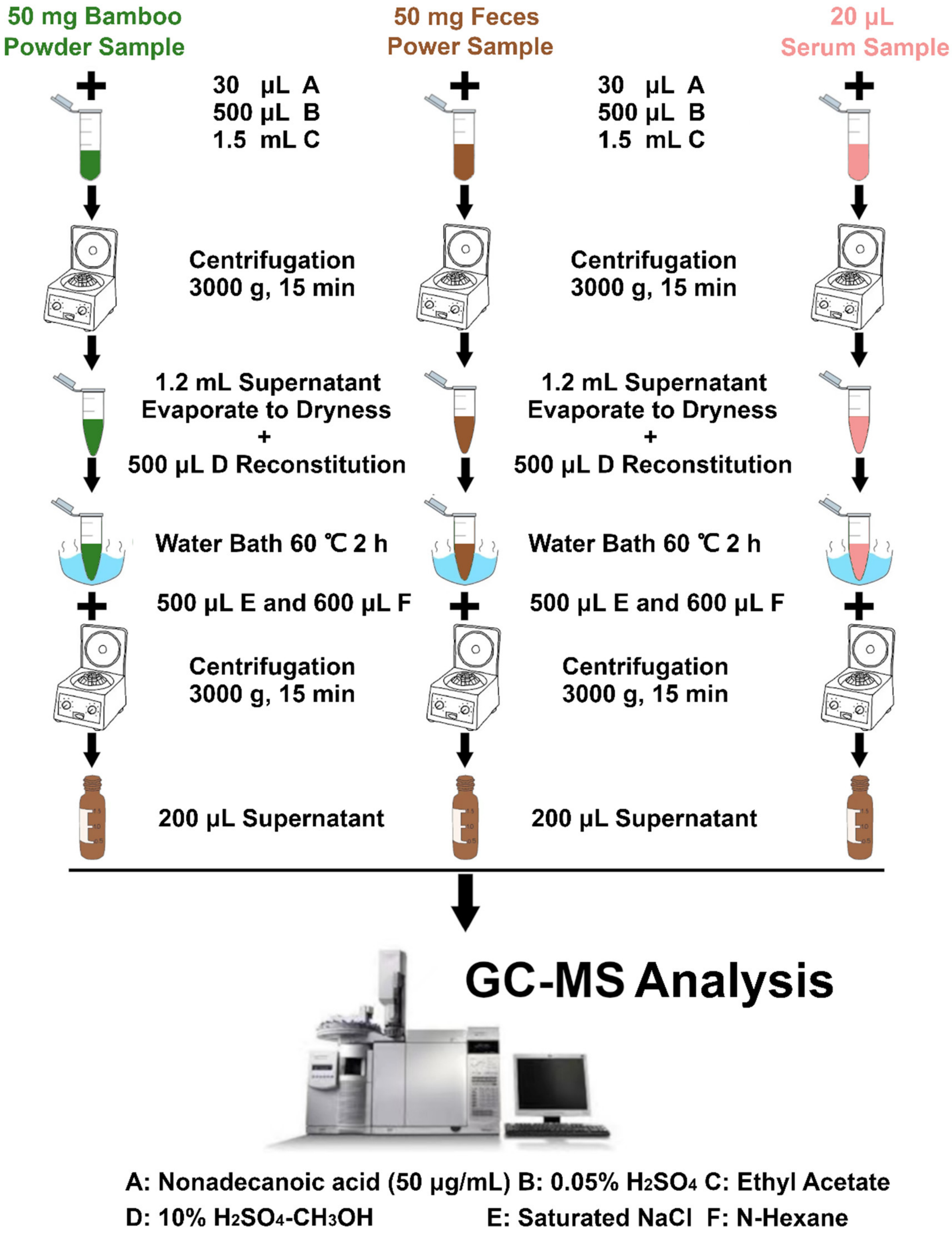

Figure S2. The workflow of sample preparation of bamboo, giant panda feces and serum using for MCFAs, LCFAs and VLCFAs quantifications by means of GC-MS.

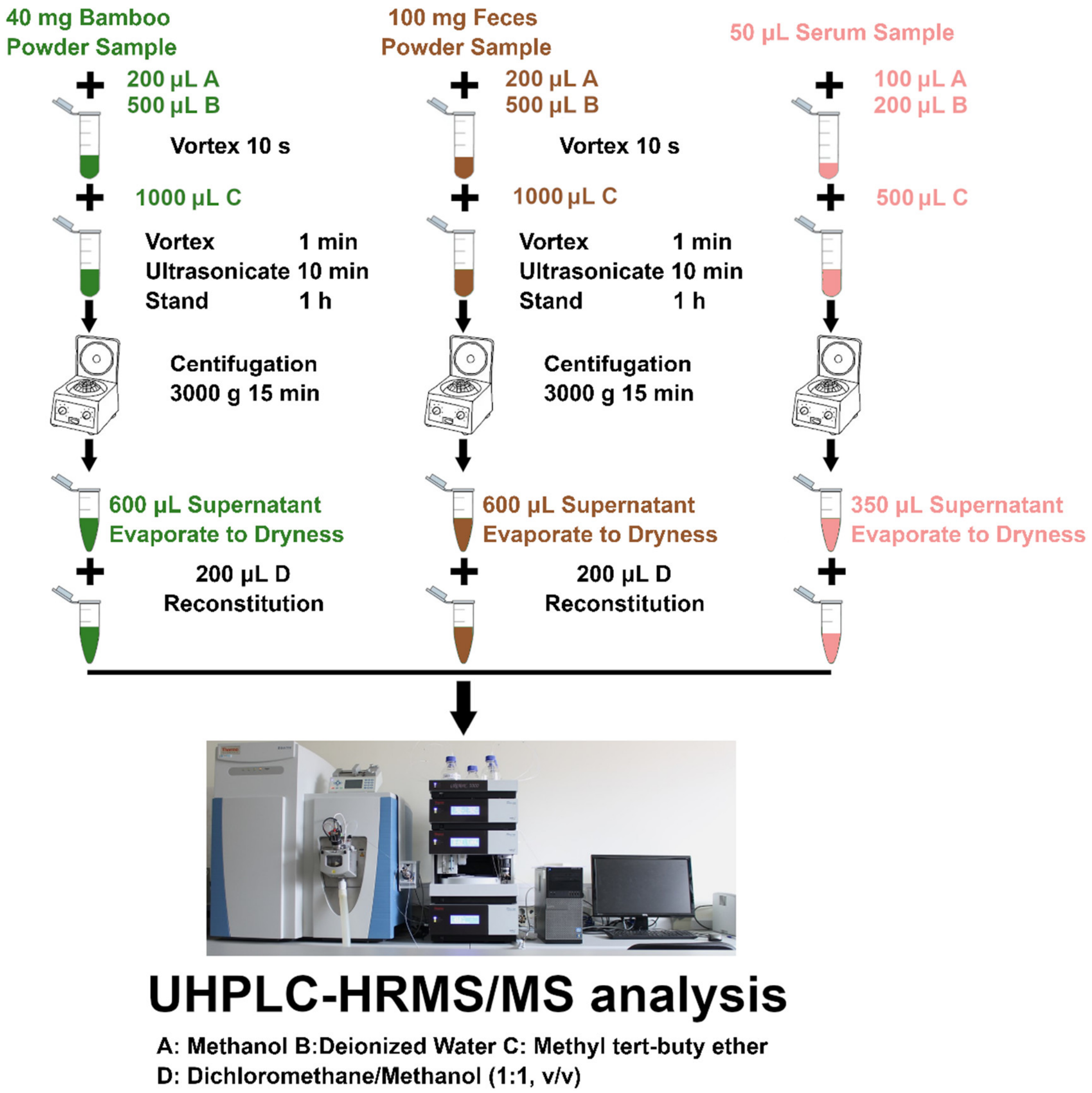

Figure S3. The workflow of sample preparation of bamboo, giant panda feces and serum using for untargeted lipidomic investigations by means of UHPLC-HRMS/MS.

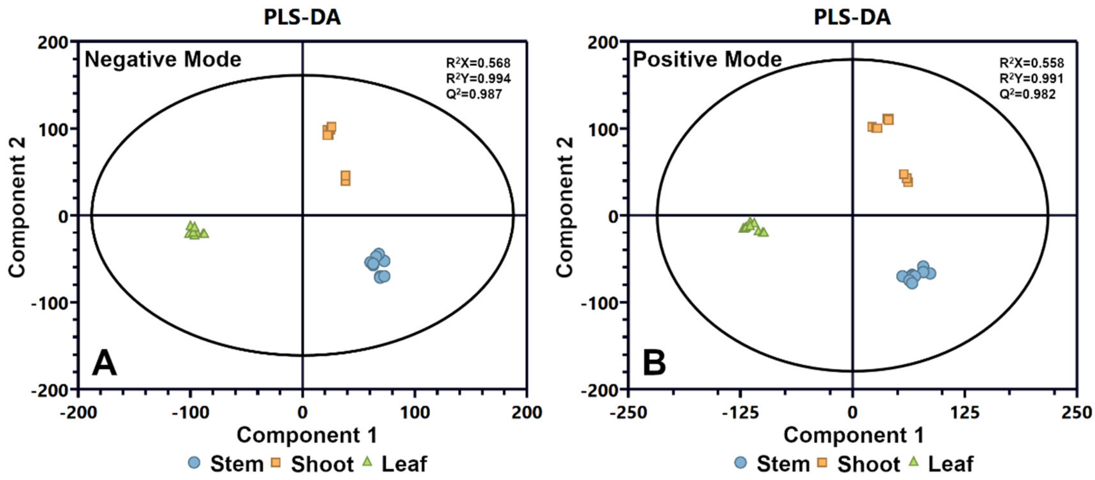

Figure S4. PLS-DA model built on the concentration of the fatty acids quantified by UHPLC-HRMS/MS in different bamboo parts.

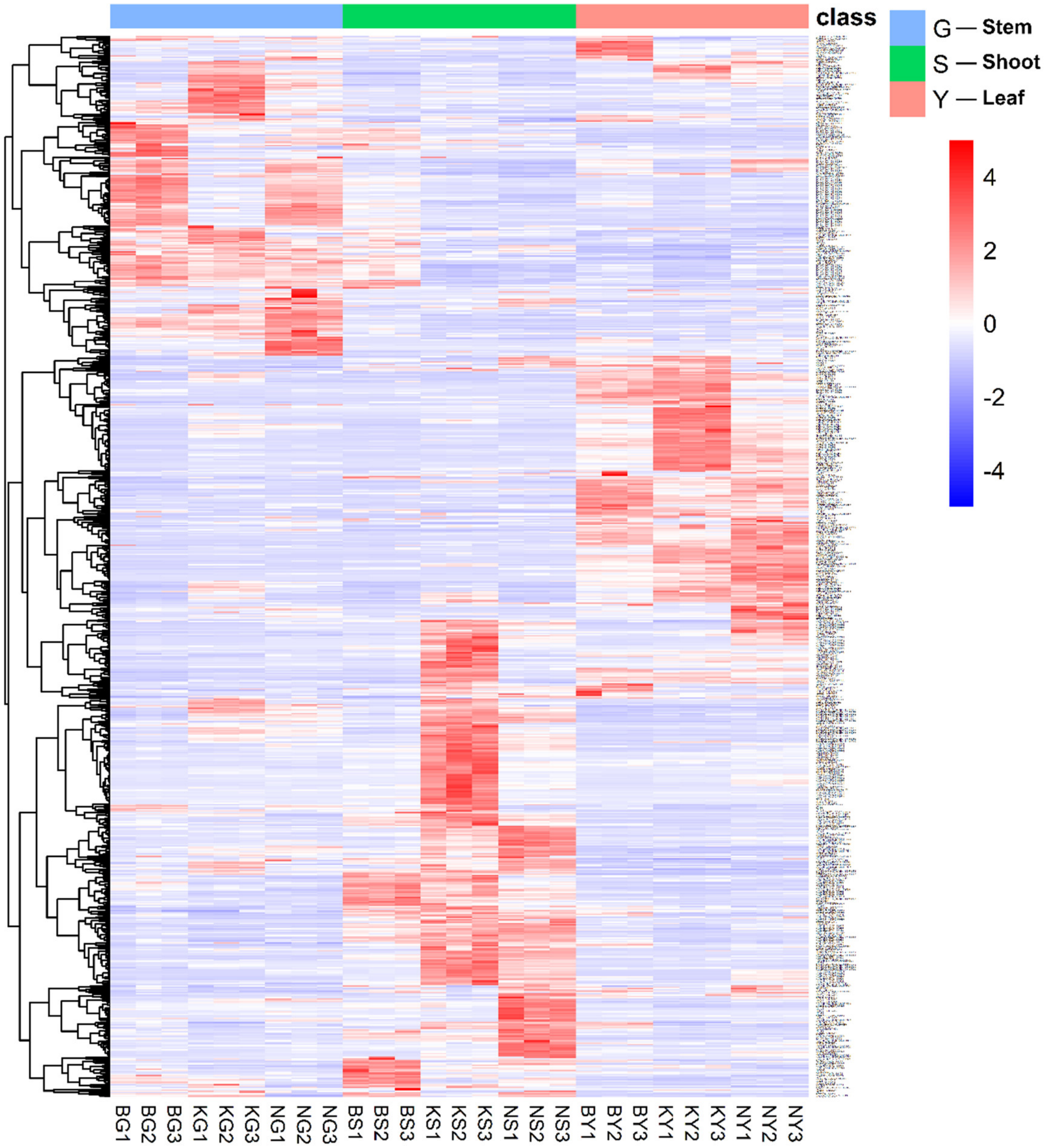

Figure S5. Heatmap of the concentration of the fatty acids quantified by UHPLC-HRMS/MS in different bamboo parts which showed a statistically significant difference among stem, shoot and leaf groups.

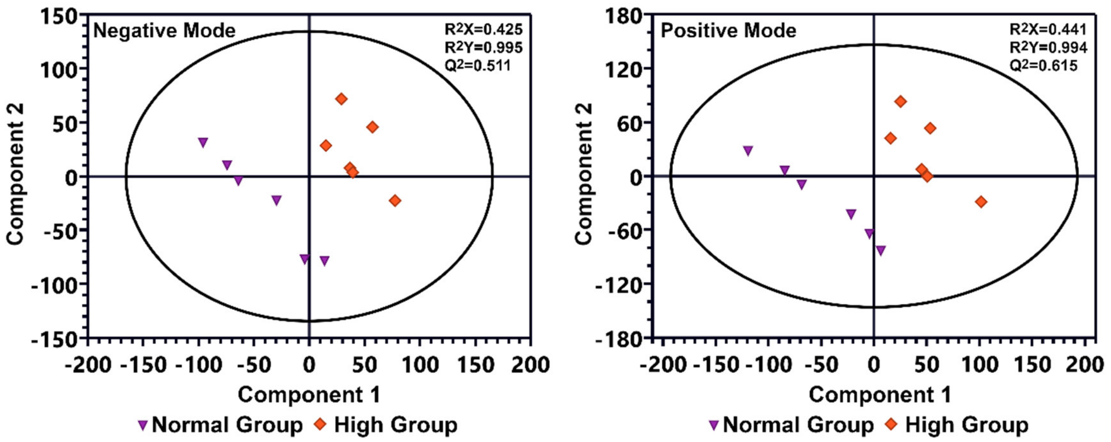

Figure S6. PLS-DA model built on the concentration of the fecal fatty acids quantified by UHPLC-HRMS/MS.

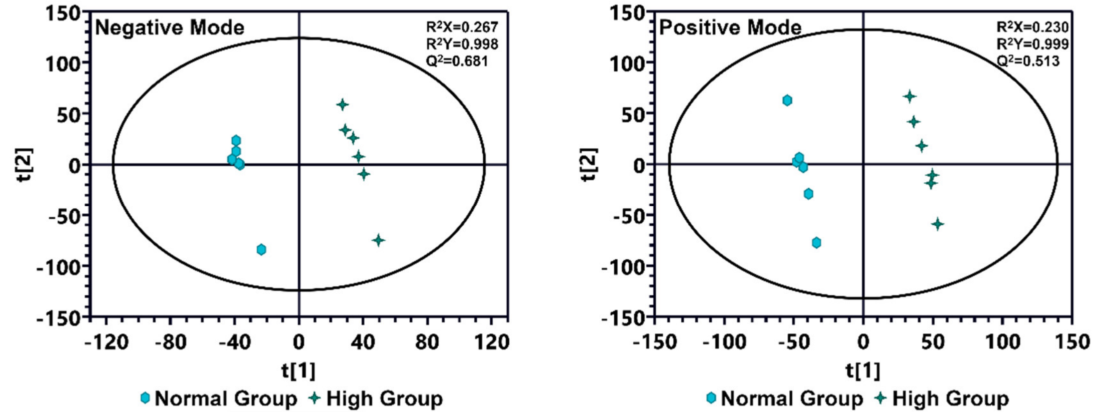

Figure S7. PLS-DA model built on the concentration of the fatty acids quantified by UHPLC-HRMS/MS in giant panda serum.

Detailed Materials and Methods

GC-MS analysis

-SCFAs An amount of 100 mg bamboo sample was mixed with 220 μL of bidistilled water, 30 μL of 0.5 M Na_2HPO_4, 50 μL of internal standard aqueous solution (100 μg /mL acetic acid-d4, propanoic acid-d6 and butyric acid-d7) and 600 μL of PFBBr acetone solution. The quantity of 100 mg of feces sample was added by 170 μL of bidistilled water and 30 μL of 0.5 M Na_2HPO_4. Then 100 μL of the above internal standard aqueous solution and the same amount of PFBBr acetone solution were mixed. For serum samples, 100 μL liquids was taken to an Eppendorf tube with 20 μL of bidistilled water and 30 μL of 0.5 M Na_2HPO_4 added before. The solution was

added by 50 μL of internal standard aqueous solution, and mixed with 400 μL of PFBBr acetone solution. Following incubation at 60 °C water bath for 40 min, 400 μL of n-hexane was added to the above solutions. Finally, all the samples were centrifugated and 50 μL n-hexane layer (upper layer) was collected prior to GC-MS analysis. The workflow of sample preparation was reported in Figure S1.

Instrumental analysis was performed on an Agilent 7890A gas chromatography system coupled to an Agilent 5975C inert MSD system (Agilent Technologies Inc., CA, USA). An OPTIMA WAXplus® fused-silica capillary column (30 m × 0.25 mm × 0.25 μm; MACHEREY-NAGEL, Düren, GER) was utilized to separate the derivatives. Helium (>99.999%) was used as a carrier gas at a constant flow rate of 1 mL/min through the column. Injection volume was 1 μL and the solvent delay time was 9 min. The initial oven temperature was held at 60 °C for 2 min, ramped to 100 °C at a rate of 20 °C/min, to 110 °C at a rate of 2 °C/min, to 160 °C at a rate of 10 °C/min, to 240 °C at a rate of 20 °C/min, and finally held at 240 °C for 2 min. The temperatures of injector, transfer line, and electron impact ion source were set to 250 °C, 250 °C, and 230 °C, respectively. The impact energy was 70 eV, and data was collected in a SIM mode.

-Median, Long and Very Long Chain Fatty Acids (MCFAs, LCFAs and VLCFAs)

For MCFAs, LCFAs and VLCFAs quantification, we used nonadecanoic acid as internal standard, which can be detected but not quantified in the samples. Therefore, the maximum potential ratio between native internal standard and added internal standard is 1.3%. According to Dolan (When Should an Internal Standard be Used? (chromatographyonline.com)), if the native internal standard peak is never more than 2% of the added internal standard peak, the results probably will show no adverse effects. Therefore, it is unlikely that there will be a problem that acid C 19 was used as internal standard in our study.

For bamboo and feces samples, they were ground into powder with liquid nitrogen. Taking 50 mg of feces and bamboo powder samples and 20 μL thawed serum sample respectively, 30 μL nonadecanoic acid (50 μg /mL) and 500 μL of 0.05% H_2SO_4 were added. Free fatty acids (FFAs) were extracted using 1.5 mL ethyl acetate. After

centrifugation at 3000 g for 15 min at room temperature, 1.2 mL upper layer (ethyl acetate) was collected and evaporated to dryness under nitrogen stream. The residue was reconstituted in 500 μL 10% H_2SO_4-CH_3OH and the resulting mixture was incubated at 60 °C water bath for 2 h. Then, 500 μL saturated NaCl and 600 μL n-hexane were added. After centrifugation, 200 μL n-hexane phase was transferred to a GC-vial prior to GC-MS analysis. The workflow of sample preparation was reported in Figure S2.

Instrumental analysis was performed on an Agilent 7890A gas chromatography system coupled to an Agilent 5975C inert MSD system (Agilent Technologies Inc., CA, USA). A J&W DB-23 fused-silica capillary column (20 m × 0.18 mm × 0.2 μm; Agilent Technologies Inc., CA, USA) was utilized to separate the derivatives. Helium (>99.999%) was used as a carrier gas at a constant flow rate of 0.9 mL/min through the column. Injection volume was 1 μL in split ratio of 2:1. The solvent delay time was 3 min. The initial oven temperature was held at 50 °C for 1 min, ramped to 175 °C at a rate of 50 °C/min, to 230 °C at 8 °C/min and finally held for 2 min. The temperatures of injector, transfer line, and electron impact ion source were set to 220 °C, 250 °C, and 230 °C, respectively. The impact energy was 70 eV, and data was collected in a full scan mode (m/z 50-600).

UHPLC-HRMS/MS analysis

Prior to analysis, bamboo and feces samples were ground to powder with liquid nitrogen. To 40 mg bamboo and 100 mg feces samples respectively, 200 μL methanol and 500 μL deionized water were added. After the mixture was vortexed for 10 s, 1000 μL MTBE (methyl tert-butyl ether) was subsequently supplemented. The mixtures were vortexed for 1 min, ultrasonicated for 10 min, and stood for 1 h at room temperature. After centrifugation at 3000 g for 15 min, 600 μL supernatant (MTBE phase) was evaporated to dryness. The residues were reconstituted in 100 μL dichloromethane/methanol (1:1, v/v) prior to perform UHPLC-HRMS/MS analysis. Quality control (QC) sample was pooled from the supernatants of all bamboo samples and feces samples respectively, and then 600 μL was evaporated to dryness. The

residues were reconstituted in 200 μL dichloromethane/methanol (1:1, v/v) prior to UHPLC-HRMS/MS analysis. To 50 μL thawed serum sample, 100 μL methanol and 200 μL deionized water were added. After the mixture was vortexed for 10 s, 500 μL MTBE (methyl tert-butyl ether) was subsequently supplemented. The mixtures were vortexed for 1 min, ultrasonicated for 10 min, and stood for 1 h at room temperature. After centrifugation at 3000 g for 15 min, 350 μL supernatant (MTBE phase) was evaporated to dryness. The residues were reconstituted in 200 μL dichloromethane/methanol (1:1, v/v) prior to perform UHPLC-HRMS/MS analysis. Quality control (QC) sample was pooled from all serum samples, was prepared as that of serum sample. The workflow of sample preparation was reported in Figure S3.

Chromatographic separation was performed on a Thermo Fisher Ultimate 3000 UHPLC system with a Waters BEH C18 column (2.1mm × 100 mm, 1.7 μm) with column temperature at 50 °C. The injection volume was 4 μL for serum sample, 5 μL for feces sample and 5 μL for bamboo sample. The mobile phases consisted of 60:40 acetonitrile:water (v/v) (phase A) and 90:10 isopropanol:acetonitrile (v/v) (phase B), both with 10 mM ammonium formate (v/v). The flow rate was 0.3 ml/min. A linear gradient elution was performed with the following program: 0 min, 30% B; 10.5 min, 100% B and held to 12.5 min; 12.51 min, 30%B and held to 15 min.

The eluents were analyzed on a ThermoFisher Q Exactive™ Hybrid Quadrupole-Orbitrap™ Mass Spectrometry (QE) in Heated Electrospray Ionization Positive (HESI+) and Negative (HESI-) mode, respectively. Spray voltage was set to 3000 V for HESI+ and 2800 V for HESI-. Capillary and Aux Gas Temperature were separately 350 °C and 350 °C. Sheath gas flow rate was 35 (Arb. Unit) for HESI+ mode, respectively. Aux gas flow rate was 15 (Arb). S-Lens RF Level was 50 (Arb. Unit). The full scan was operated at a high-resolution of 70000 FWHM (m/z=200) at a range of 250 - 1500 m/z with AGC Target setting at 1×10^6. Simultaneously, the fragment ions information of top 10 precursors each scan was acquired by Data-dependant acquisition (DDA) with HCD energy at 15, 25 and35 eV, mass resolution of 17500 FWHM, and AGC Target of 2×105.
